# Projected and Observed Aridity and Climate Change in the East Coast of South India under RCP 4.5

**DOI:** 10.1155/2015/169761

**Published:** 2015-12-03

**Authors:** A. Ramachandran, Dhanya Praveen, R. Jaganathan, K. Palanivelu

**Affiliations:** ^1^Centre for Climate Change and Adaptation Research, Anna University, Guindy, Chennai 600025, India; ^2^Tamil Nadu Agricultural University, Coimbatore, Tamil Nadu 641003, India

## Abstract

In the purview of global warming, the present study attempts to project changes in climate and quantify the changes in aridity of two coastal districts in south India under the RCP 4.5 trajectory. Projected climate change output generated by RegCM 4.4 model, pertaining to 14 grid points located within the study area, was analyzed and processed for this purpose. The meteorological parameters temperature and precipitations were used to create De Martonne Aridity Index, to assess the spatial distribution of aridity. The original index values ranged from 13.7 to 16.4 mm/°C, characterizing this area as a semidry climate. The outcome from the changed scenario analysis under RCP 4.5 showed that, during the end of the 21st century, the aridity may be increased more as the index values tend to reduce. The increasing trend in the drying phenomenon may be attributed to the rising of mean annual temperatures.

## 1. Introduction

Altering climate is a complex phenomenon which has intricate implications. Arid and semiarid regions of the world are highly sensitive to human-induced climate and/or land transformation [1]. Assessing the changing patterns of aridity is a serious matter of concern in the context of global climate change. The studies have indicated that, over the preceding 20 years, the problem of land degradation continued to worsen due to climate change [2]. The Intergovernmental Panel on Climate Change reported increasing frequency of dry spells and drought can be serious consequence of climate change [3]. The arid and semiarid regions comprise almost 40% of the world's land surfaces. These sites, which are currently at the limit with respect to available water resources, are likely to be most sensitive to climate change [4, 5]. There is a high degree of certainty that global climate change, land use developments, and land cover changes will lead to an accelerated decline in water availability and biological production in dry lands [3, 6]. The latest estimates of future climate change predict that there will be an increment of 5–10 days in the consecutive dry days as per RCP 8.5 emission pathways for the period 2081–2100 for India [7]. In recent years, semiarid tracts of India have been experiencing growing water scarcity that can be mainly related to climate variations and poor soil and water management.

Aridity is the degree to which a climate lacks effective, life-promoting moisture; it is the opposite of humidity, in the climate sense of the term [8]. In the case of aridity, the lack of rainfall depends on the local climate and represents a permanent or seasonal condition. Researchers have focused on drought and extensively reported on aridity issues in particular throughout the world [9–12]. As per the future projections, the arid and semiarid regions are going to be impacted severely through heavy land degradations, loss of biodiversity, and food insecurities [13]. Water is going to be the major limiting factor for the healthy functioning of ecosystems, especially agriculture production worldwide [14, 15]. Land degradation means reduction of or loss in the biological or economic productivity and complexity of rain-fed cropland, irrigated cropland, range, pasture, forest, or woodlands resulting from land uses or from processes arising from human activities and habitation patterns [16]. The weather conditions and the climate of a region constitute basic factors for the growth and development of plants. The change in the per capita area of arable land in India from the period 1950–55 to 2000 is 0.9 to 0.15 ha and projected to decline by 0.08 to 0.07 ha by the year 2025 [7]. This increased water stress will lead to reduced productivity of croplands and availability of fresh water, resulting in further adverse impacts on human well-being in dry lands. Scientific reports have already indicated that global warming is likely to pose a defining challenge on most of the economic sectors in India that are driven by climate such as water resources, agriculture and allied services, biodiversity, and forests [17].

In this context, the present study focuses on projecting the changes in the spatial distribution of climate and aridity conditions of two coastal districts of South India using De Martonne aridity indices that determine the climatic conditions of a place based on temperature and precipitation. There are numerous climatic indices available using precipitation values alone; however, this index takes into account both the precipitation and the temperature conditions of an area. In the emerging scenario of global warming, this index is the appropriate one for characterizing and projecting future aridity conditions. It has been proven to be very useful for planning and managing agricultural production. This research work would shed some light on the need to have aridity projections at local, regional, and global scales in order to identify the most affected areas and most vulnerable ecosystems and population.

### 1.1. Profile of the Study Area

**Figure 1:**
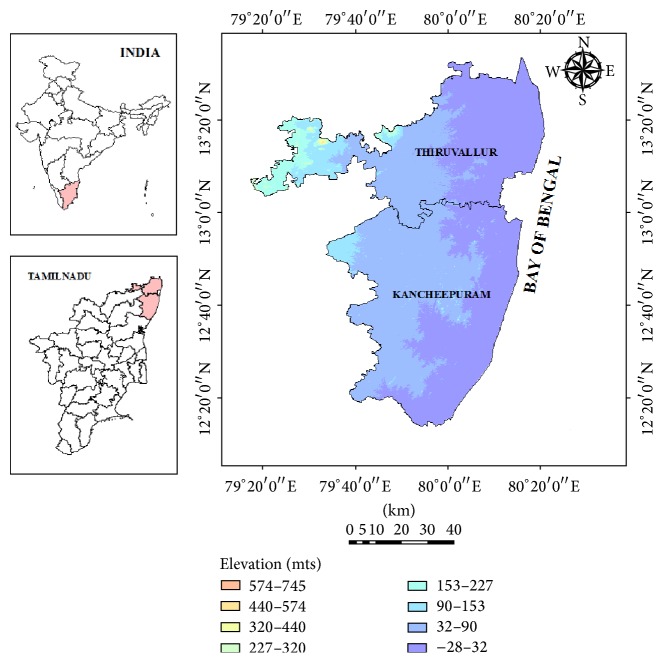
Location map of the study area with the RegCM grid points.

The study region, Chengalpet (erstwhile known by this name), covers present Thiruvallur and Kancheepuram coastal districts of Tamil Nadu, South India (Figure 1). It is located in the northeast agroclimatic zones of Tamil Nadu between the latitudes 12°0′ and 13°40′N and 79°0′ to 80°20′E longitude. It lies on the northeast coasts of Tamil Nadu and is flanked by the Bay of Bengal to its east with a coastline of 115.1 kms and located adjacent to Chennai Metropolitan City, the fourth largest metropolis in India. The general slope of the study area is from northwest to southeast. The elevation of this area is within 100–200 meters above MSL.

The interior part of this region is categorized as tropical semiarid while the coastal parts are categorized as dry subhumid climate. Under the Drought Prone Area Programme, Kancheepuram and Thiruvallur Districts come under the semiarid category of dry land classification [18]. This area receives rain under the influence of both southwest and northeast monsoons. Most of the rain obtained is in the northeast monsoon season (after monsoon) due to cyclonic storms caused by the depressions in Bay of Bengal, chiefly during October to December months. The rainfall is highly erratic. The general drainage course of the study area is from west to east coast and drains into the Bay of Bengal. The major rivers flowing within Thiruvallur District are Araniyar, Kosasthalaiyar, and Cooum rivers. The major rivers flowing through Kancheepuram areas are Palar, Cheyyur, Killiyar, and Vegavathi rivers. Another major drainage is Buckingham canal, which runs from north to south and ends into the sea after merging with the Palar River. These rivers are seasonal and carry substantial flows during the monsoon period. The chief irrigation sources in the area are the tanks, wells, tube wells, and canals. The maximum daily temperature rarely exceeds 43°C and minimum daily temperatures seldom fall below 18°C. The annual mean rainfall of this area is 1202 mm and it has potential evapotranspiration of 1750 mm. The rainfall in this region shows remarkable annual variability. These areas were known as “*Erie Mavattam*” (Lake District) earlier as the ancestors had constructed a series of tanks across the river basins and had good practices for harvesting the rainwater for irrigation purposes.

## 2. Experimental Section

### 2.1. Data Analysis and Models

#### 2.1.1. RegCM: Regional Climate Model

This study employed dynamical downscaling approaches using Regional Climate Model (RegCM) 4.4 rc22, with HadGEM 2-ES lateral boundary conditions. Regional Climate Model Version 4.4 (RegCM4.4) is developed by Abdus Salam International Centre for Theoretical Physics (ICTP), Italy, to simulate the future climate. It is an earth system model. For simulating the plausible future climate, the latest available emission trajectory Representative Concentration Pathway 4.5 (RCP 4.5) proposed by the AR5 report of IPCC has been used. The RCP 4.5 is said to be in good coherence with the observed climate of Indian subcontinent and was chosen for simulation [19].

#### 2.1.2. “De Martonne” Aridity Index

For accomplishing the intended purposes of this study to characterize and project the aridity situations prevailing in the study area, “*De Martonne*” Aridity Index was employed using the simulated climatic output of RegCM under RCP 4.5. Aridity is being delineated for yearly time scale values using the mean precipitation and air temperature for the 14 grids located in the study area. The projected aridity was calculated for the near- (2010–2040), mid- (2041–2070), end-century (2071–2098) periods.

RegCM simulated daily outputs of mean air maximum, minimum temperatures, and precipitation under the RCP 4.5 emission pathway at 14 grid points were processed for analysis and mapping purpose. The cover period of study was separated into baseline (1971–2000), near-century (2010–2040), mid-century (2041–2070), and end-century (2071–2100) periods. Zoning and changes in aridity maps were prepared using Arc GIS tools.

De Martonne aridity index (DM_I_) was calculated using the formula (Table 1)

(1) $$\begin{array}{c} {DM}_{I}=\frac{P}{T+10}, \end{array}$$

where *P* is the annual precipitation sum [mm] and *T* is the annual mean air temperature [°C].

## 3. Results and Discussion

### 3.1. Projected Climate Change

**Figure 2:**
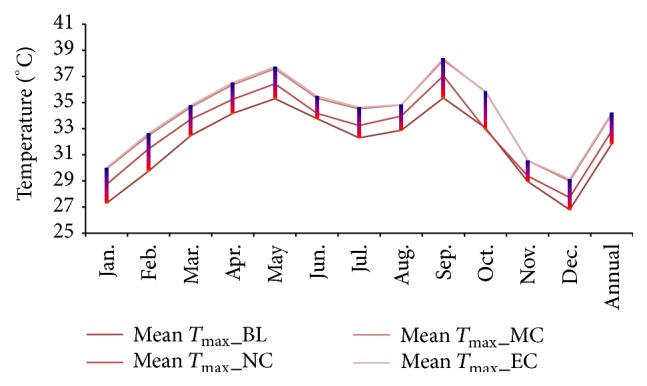
Monthly and annual variations in projected the MMax*T* in baseline, near-century, mid-century, and end-century.

**Figure 3:**
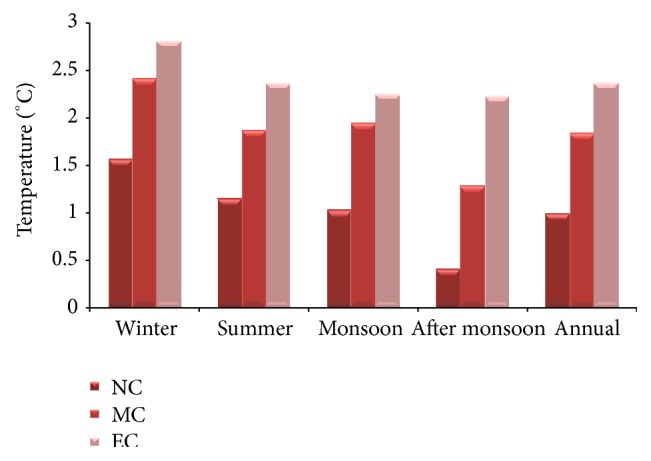
Seasonal variations in the projected MMax*T* in the baseline, near-century, mid-century, and end-century.

**Figure 4:**
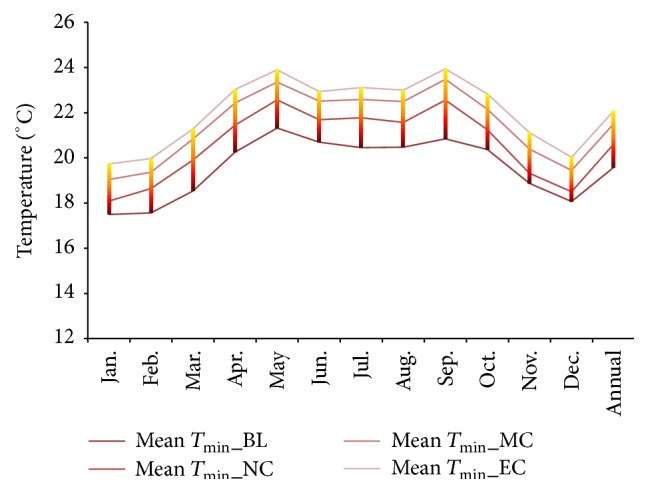
Monthly and annual variations in projected the MMin*T* in the baseline, near-century, mid-century, and end-century.

**Figure 5:**
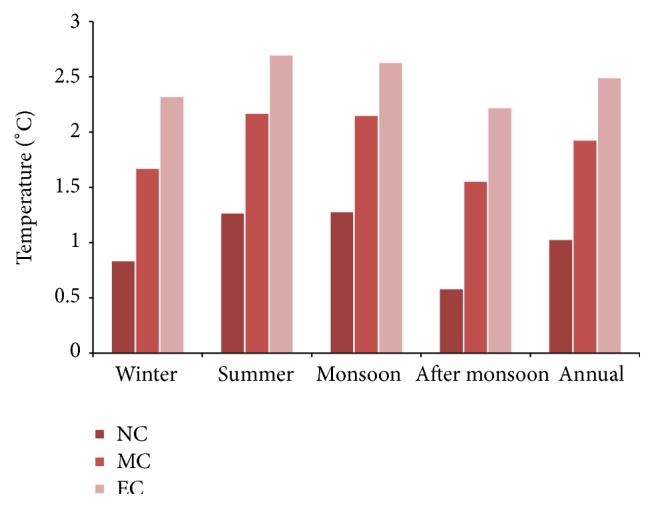
Seasonal variations in the projected MMin*T* in the baseline, near-century, mid-century, and end-century.

Projected changes in climate in the study area under RCP 4.5 showed future warming in the study region in terms of both daytime and night time temperatures. Both the simulated mean maximum and minimum temperatures showed significant warming (Table 2). The projections showed a rise in maximum temperature of 2.4°C under RCP 4.5 by the end of 21st century (Figure 2). The annual mean minimum temperature was projected to rise by 2.3°C by the near-, mid-, and end-century (Figure 3). The projections made by other researchers have shown similar trend of future warming [19–21]. The latest simulation study reported by Chaturvedi et al. [19] indicated a rise in temperature in the range of 2.9°C to 3.3°C under RCP 4.5 and RCP 6 pathways. Winter months (January and February) are projected to warm more than other months (Figures 4 and 5). This change was seen more pronounced from baseline to near-century period (Figures 2 and 3).

### 3.2. Temperature

The projected rise in daytime temperature showed the greatest increase throughout the winter season during the last part of the 21st century of around 2.8°C, followed by 2.4°C during mid-century and 1.5°C during near-century. Annually, the projections showed an increase of 2.3°C. During postmonsoon season, the daytime temperature may rise by 2.2°C in the end-century period (Figure 5).

The nighttime temperature during near- and mid-century showed higher increase. Annually, the night time temperature increased more than the daytime temperature. During the end-century period, the projected warming would be in the range 2.5°C. The highest projected warming during the summer and monsoon period is about 2.7°C and 2.6°C, respectively.

### 3.3. Rainfall

**Figure 6:**
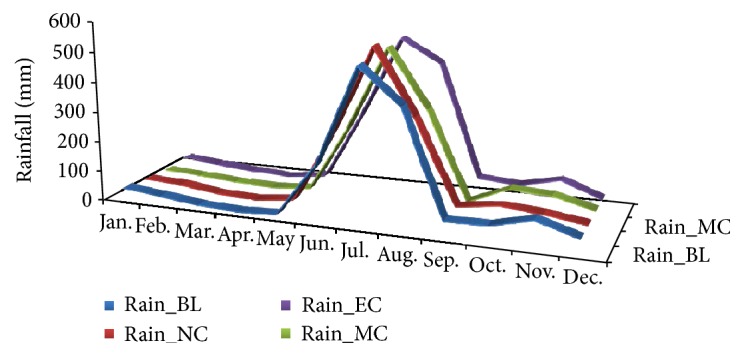
Monthly and annual variations in the projected rainfall in the baseline, near-century, mid-century, and end-century.

**Figure 7:**
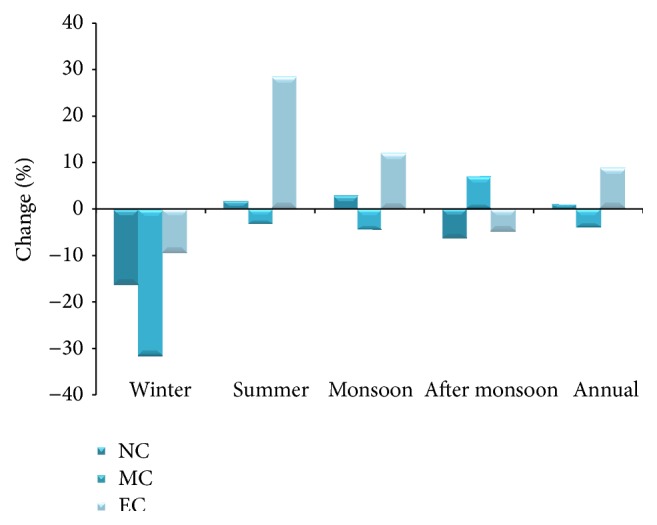
Seasonal variations in the projected rainfall in the baseline, near-century, mid-century, and end-century.

*The* Projected rainfall shows maximum decline (−31.63%) during the mid-century winter season, followed by −16% in the near-century and −9.6% in the end-century (Figures 6 and 7). Projected summer rainfall at the end-century showed 28% increase compared to the baseline period. Monsoon rainfall showed a likely decline of 4% around mid-century and there after showed an increase of 11% from baseline period. Northeast monsoon rainfall tends to increase slightly in the mid-century and there after shows decrease. The annual rainfall, on the other hand, shows slight decrease in the mid-century and there after increased by 9%. This variations would significantly impact the cropping pattern as well as the agriculture productivity.

The spatial distributions of MMax*T* and MMin*T* showed an apparent increase towards the end-century period. Under the future RCP 4.5 pathways, RegCM model had projected a rise in both MMax*T* and MMin*T* over the study area with a clear increase of 2.3°C and 2.5°C for the end century (2070–2098) period, respectively.

**Figure 8:**
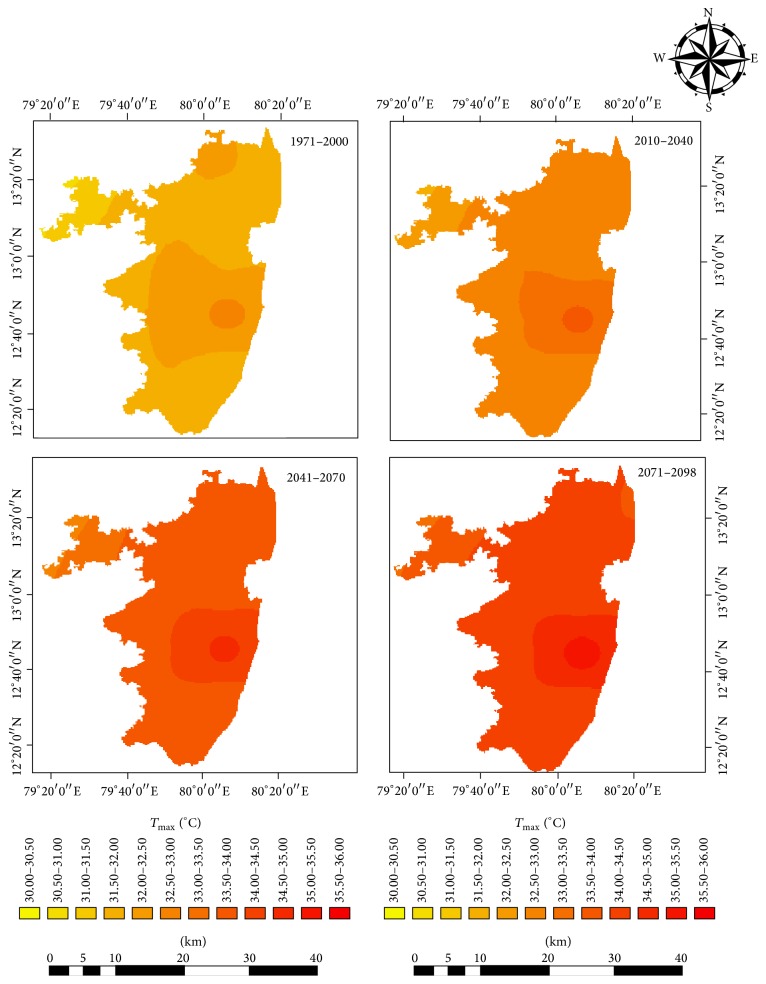
Spatial distribution of projected MMax*T* in the baseline, near-century, mid-century, and end-century.

**Figure 9:**
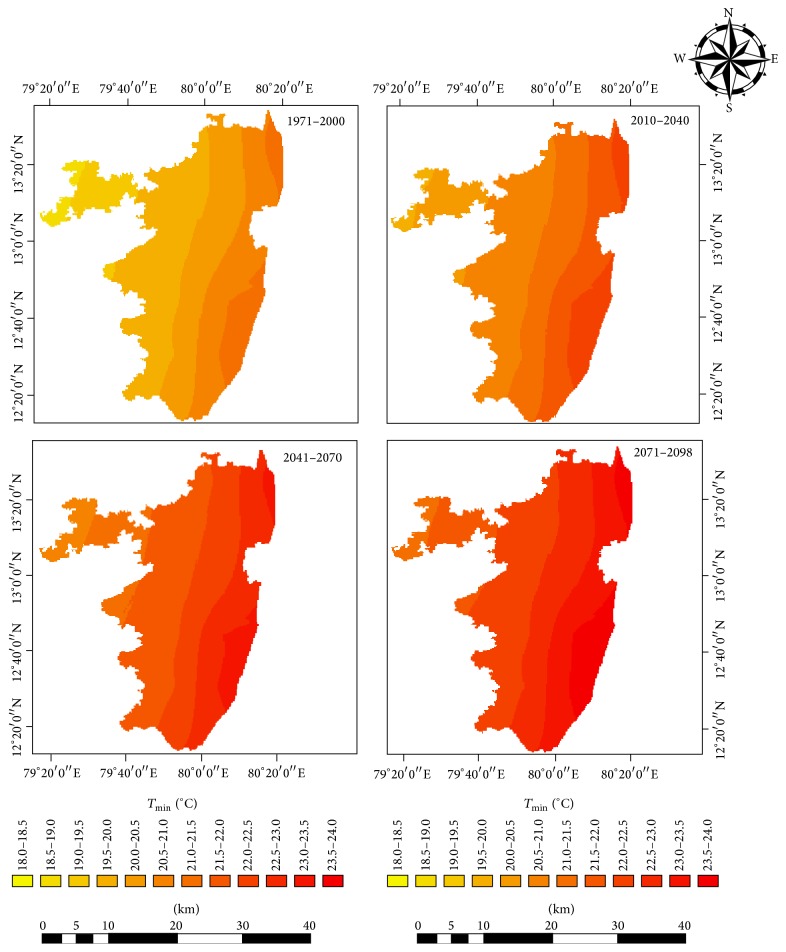
Spatial distribution of projected MMin*T* in the baseline, near-century, mid-century, and end-century.

Under the future RCP 4.5 pathways, the spatial distributions of MMax*T* and MMin*T* and rainfall are shown in the Figures 8 and 9. Notable variations were observed spatially with an apparent increase towards the end of the 21st century. The increases in temperature were found to be more severe towards the coastal areas than the interiors. The areas near Chennai City had more rapid warming than the other parts. Future projections of climate using regional climate models PRECIS and RegCM 3 under A1B scenario have projected an increase in maximum and minimum temperature by 3.1 to 3.7°C and 3.7 to 4.2°C, respectively, at the end of the 21 century for Tamil Nadu as compared to the baseline period 1970 to 2000.

Many researchers around the world have investigated the impact of expected global climate change on regional water availability. Employing the regional climate model ECHAM4 and the results reveals a clear increase of mean annual temperature of 1.2°C–1.3°C for the Volta region [8, 22].

**Figure 10:**
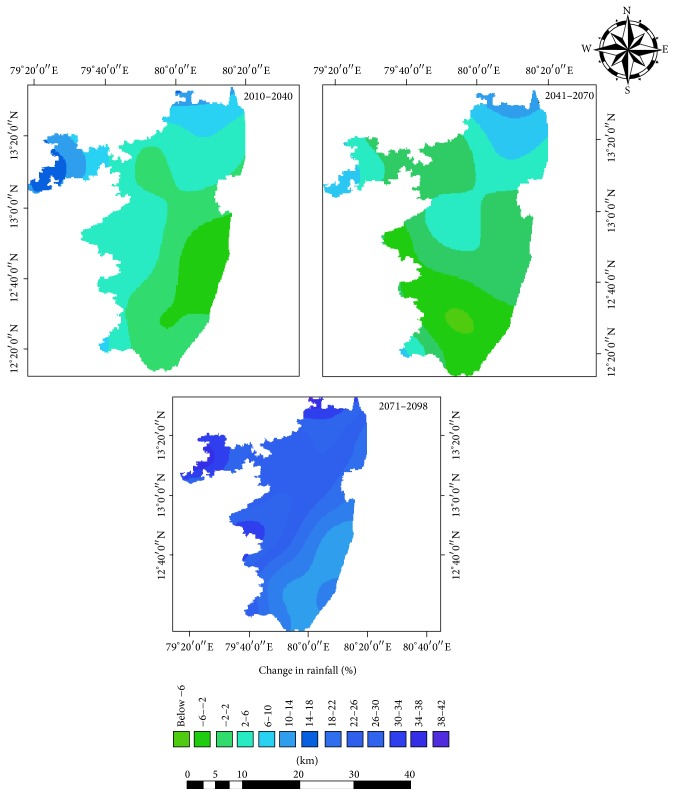
Spatial distribution of projected rainfall in the baseline, near-century, mid-century, and end-century.

As far as rainfall projections were concerned, during near- and mid-century periods, the southeastern parts of the study area, covering Tirukalukundram, Latur, Thiruporur, and Madurantakam, were likely to have a decrease in rainfall of 2 to 6% compared to the baseline period (Figure 10). The western and northwestern parts were projected to receive increased rainfall towards the end of the 21st century.

**Figure 11:**
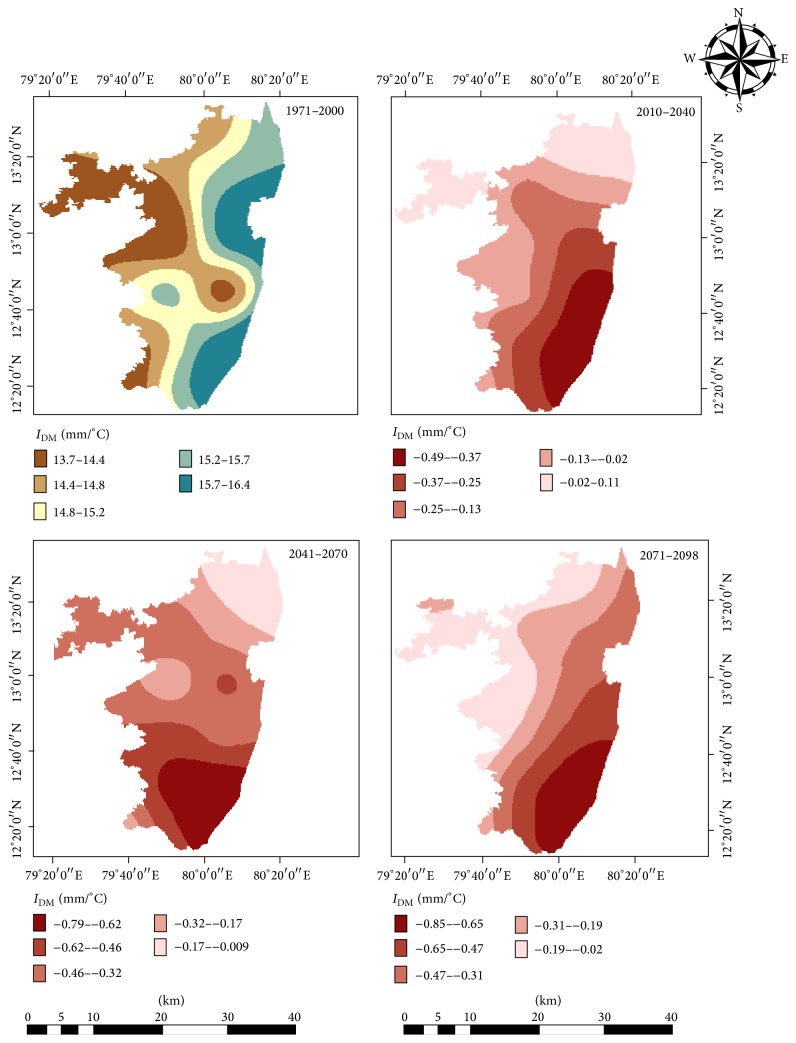
Projected changes in aridity in baseline period (1971–2000), near-century (2010–2040), mid-century (2040–2070), and end-century (2070–2100).

The De Martonne Aridity Index was calculated by processing and analyzing the RegCM simulated air temperature and precipitation data of the study using 14 grid points for various time slices (Figure 1).  Figure 11 depicts the projected mean annual aridity index for 30-year periods for near-, mid-, and end-century along with the baseline period (1970–2000). An apparent drying trend was observed towards the end of the 21st century as there was a decline in the index values. The original mean index values ranged from 13.7 to 16.4 mm/°C, characterizing this area already under semiarid dry climate. As the index values ranged between 13 and 14 mm/°C, the northwestern parts of the study covering parts of Tiruttani, Tiruvalankadu, R.K.Pet, and Pallipattu areas are already experiencing dry climate. But projections showed that southeastern coastal stretch may experience more drying trends under the changed climatic conditions.

The spatial distribution of the De Martonne Aridity Index represented in Figure 11 denotes that semiarid climate is prevailing in the study area. In particular, Latur, Tirukalukundram, and Chithamur administrative blocks may likely face more dryness in future as the projected aridity values tend to decline, indicating more drying trends over these areas under the RCP 4.5 emission scenario. There was a clear variation in dryness noted from the coastal stretch to interior parts. The values of the index seemed to increase gradually from the western part of the study area to east coastal regions. This is in line with the spatial distribution of precipitation and temperature as this index takes into account both these prime climate variables. For this reason, the application of De Martonne Index led to a more precise characterization of each grid point taken for the entire analysis.

The scale of climate change is of great concern, and the observed trends and anticipated consequences of accelerated dry spells, hot days, and so forth portend a series of threats in the future for the dependent communities. More frequent hot days, hot nights, and heat waves have also been noticed in many parts of India [18–21]. The latest advancements in global and regional climate simulation models helped us to better understand and project future climate changes, based on different emission scenarios and trajectories [23].

Meteorological parameters were often used to create specific climate indices to assess the temporal trend of aridity such as De Martonne Aridity Index, UNEP Aridity Index, and Water Deficit Index, which are among the most representative of the analyzed phenomenon. Many researchers around the world have analyzed the annual and seasonal precipitation series and carried out annual aridity indices. Their findings reveal that the sites which are currently at the limit with respect to available water resources in semiarid regions are likely to be most sensitive to climate change [23–26]. Prăvălie [25] attempted to quantify the trend of climate aridification of southern Oltenia in the last five decades (from 1961 to 2009) using temperature, precipitations, and potential evapotranspiration. He concluded that the aridity trend is pronounced after 1980s among the five decades analyzed. Growing aridity conditions are caused primarily by the lowering of the average annual rainfall, the rising of mean annual temperatures, and the increasing of potential evapotranspiration as a result of the annual thermal regime change after 1980.

De Martonne's Index (Iar-DM) was applied to understand aridity status and to determine its relationship with irrigation water requirements of representative crops of Romania by Paltineanu et al. [26]. The results revealed high variability with the lowest values (<20 mm°C^−1^) for the driest periods in the southeastern areas of Romania. Irrigation is usually applied in the regions with Iar-DM values of 15–35 mm°C^−1^. Croitoru et al. [27, 28] have also employed aridity indices, De Martonne aridity Index (DM_I_), and the Pinna Combinative Index (IP) in order to identify critical areas in the most important agricultural regions of Romania that are prone to aridity. They used data recorded in 30 locations in the extra-Carpathian areas of Romania from 1961 to 2007. The monthly, seasonal, annual, winter wheat, and maize-growing season datasets of DM_I_ and annual values of IP were also calculated. They also identified the trends using the Mann-Kendall test and Sen's slope, while the ordinary Kriging technique was employed for interpolation. Their main findings were that the most vulnerable areas to semiaridity are the southeastern regions and they reported that the usage of DM_I_ is more appropriate for this kind of assessments compared to IP index. It can be concluded that some terrestrial ecosystems are highly prone to rise in aridity and resultant dryness in response to the warming climatic indications.

## 4. Conclusions

This research has shown that warming is pronounced in the study region and indicates a notable day time and night time warming of 2.3°C and 2.5°C in future under RCP 4.5. An apparent drying trend was observed with a gradient from northwestern to southeastern parts of the study area towards the end of the 21st century as there was a decline in the De Martonne Aridity index values. The index values for the baseline period ranged from 13.7 to 16.4 mm/°C, characterizing this area under semiarid climate. Similar studies using higher resolution projection data would provide a clearer picture at subdistrict level. Microlevel studies are the need of the hour as there exists spatial variability in climatic conditions and to design local level planning. It is very indispensable for India to generate observed data at microlevel and make it available to the scientific research community to validate these kinds of studies. This would worsen the already existing acute water scarcity issues of this area in general and may aggravate the drying of rivers, lakes, and farm ponds and these areas may become not suitable for irrigated agriculture practices in the future. It is a crucial call that these scientific inputs need to be incorporated while framing local policies.

## Figures and Tables

**Table 1:** Aridity classes as per De' Martonne Index (DM_I_) classification.

Climate	Values of DM_I_
Dry	DM_I_ < 10
Semidry	10 ≤ DM_I_ ≤ 20
Mediterranean	20 ≤ DM_I_ < 24
Semihumid	24 ≤ DM_I_ < 28
Humid	28 ≤ DM_I_ < 35
Very humid	(a) 35 ≤ DM_I_ ≤ 55 (b) DM_I_ > 55

**Table 2:** Variations in the projected mean climate in the baseline, near-century, mid-century, and end-century.

Time slice	*T*_max_ (°C)	*T*_min_ (°C)	RF (%)
2011–2040	0.961	1.048	0.997
2040–2070	1.852	1.950	−16.902
2071–2098	2.331	2.503	24.906
